# Implementation research to increase treatment coverage of possible serious bacterial infections in young infants when a referral is not feasible: lessons learnt

**DOI:** 10.1093/pubmed/fdab409

**Published:** 2022-02-09

**Authors:** Shabina Ariff, Sajid Bashir Soofi, Zamir Suhag, Suhail Chanar, Maria Bhura, Zaib Dahar, Imran Ahmed, Ali Turab, Atif Habib, Yasir Bin Nisar, Samira Aboubaker, Steve Wall, Abdul Wahab Soomro, Shamim Ahmad Qazi, Rajiv Bahl, Zulfiqar A Bhutta

**Affiliations:** Department of Paediatrics and Child Health, Aga Khan University, Karachi, Pakistan; Department of Paediatrics and Child Health, Aga Khan University, Karachi, Pakistan; Centre of Excellence in Women & Child Health, Aga Khan University, Karachi, Pakistan; People’s Primary Healthcare Initiative, Sindh, Pakistan; Centre of Excellence in Women & Child Health, Aga Khan University, Karachi, Pakistan; Centre of Excellence in Women & Child Health, Aga Khan University, Karachi, Pakistan; People’s Primary Healthcare Initiative, Sindh, Pakistan; Centre of Excellence in Women & Child Health, Aga Khan University, Karachi, Pakistan; Centre of Excellence in Women & Child Health, Aga Khan University, Karachi, Pakistan; Centre of Excellence in Women & Child Health, Aga Khan University, Karachi, Pakistan; Department of Maternal Newborn Child, Adolescent Health and Aging, World Health Organization, Geneva, Switzerland; Department of Maternal Newborn Child, Adolescent Health and Aging, World Health Organization, Geneva, Switzerland; Saving Newborn Lives, Save the Children, Washington DC, USA; People’s Primary Healthcare Initiative, Sindh, Pakistan; Former WHO Staff, Geneva, Switzerland; Department of Maternal Newborn Child, Adolescent Health and Aging, World Health Organization, Geneva, Switzerland; Centre of Excellence in Women & Child Health, Aga Khan University, Karachi, Pakistan

**Keywords:** basic health unit, implementation research, lady health worker, possible serious bacterial infection, sick young infants, simplified antibiotic regimens

## Abstract

**Background:**

The objective was to achieve high coverage of possible serious bacterial infections (PSBI) treatment using the World Health Organization (WHO) guideline for managing it on an outpatient basis when referral to a hospital is not feasible.

**Methods:**

We implemented this guideline in the programme settings at 10 Basic Health Units (BHU) in two rural districts of Sindh in Pakistan using implementation research. A Technical Support Unit supported the programme to operationalize guidelines, built capacity of health workers through training, monitored their clinical skills, mentored them and assured quality. The community-based health workers visited households to identify sick infants and referred them to the nearest BHU for further management. The research team collected data.

**Results:**

Of 17 600 identified livebirths, 1860 young infants with any sign of PSBI sought care at BHUs and 1113 (59.8%) were brought by families. We achieved treatment coverage of 95%, assuming an estimated 10% incidence of PSBI in the first 2 months of life and that 10% of young infants came from outside the study catchment area. All 923 infants (49%; 923/1860) 7–59 days old with only fast breathing (pneumonia) treated with outpatient oral amoxicillin were cured. Hospital referral was refused by 83.4% (781/937) families who accepted outpatient treatment; 92.2% (720/781) were cured and 0.8% (6/781) died. Twelve (7.6%; 12/156) died among those treated in a hospital.

**Conclusion:**

It is feasible to achieve high coverage by implementing WHO PSBI management guidelines in a programmatic setting when a referral is not feasible.

## Introduction

Severe infections, referred to as possible serious bacterial infections (PSBI), in the neonatal and young infant up to 2 months of age are a global health issue and a major cause of mortality, contributing to more than a third of deaths in this age group.^1^ An estimated 6.9 million newborns were identified and treated for PSBI each year in sub-Saharan Africa, Latin America and South Asia, and over 500 000 deaths were reported globally.^2^^,^^3^ The incidence of PSBI reported in the urban settings of Pakistan is ~112 per 1000 live births.^1^

**Fig. 1:**
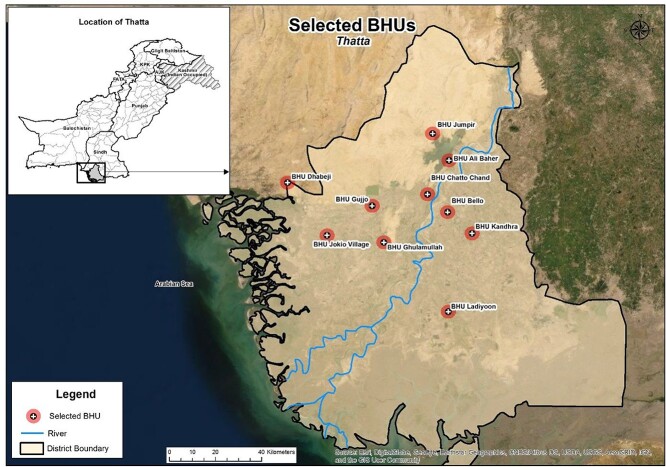
Map of the study area.

The standard management of PSBI in young infants as per the World Health Organization (WHO) guidelines is inpatient care using injectable antibiotics (penicillin or ampicillin plus gentamicin for 7–10 days), with supportive care when required.^4^ However, the guideline is not feasible for resource-limited settings, including parts of Pakistan, where refusal to hospital referral may be as high as 75%.^5^^,^^6^ Limited accessibility, affordability and poor quality of health services are some of the major reasons for not accepting a referral to a hospital.^7^

To address this issue, the WHO developed a guideline in 2015 on the management of PSBI in young infants when a referral is not feasible^8^ based on the evidence derived from clinical trials carried out in Africa and Asia.^7^^,^^9–12^ To effectively scale up this guideline in the country, it was essential to evaluate the feasibility of its implementation in the current programme settings. We implemented the WHO PSBI guideline at the primary health care (PHC) level within the country’s health infrastructure to young infants whose families refused referral to a hospital. The overall objective was to achieve high coverage of PSBI treatment and evaluate enablers and barriers to implementation. The specific aims were to demonstrate the following programme targets in early implementation: (i) all health facilities will provide simplified outpatient management of PSBI when a referral is not feasible, (ii) 80% of sick young infants will be identified and will receive treatment and (iii) 80% of the sick young infants treated will receive adequate quality treatment.

## Methods

### Study design and setting

This implementation research was a collaboration between the Aga Khan University Pakistan (AKU), People’s Primary Health Care Initiative (PPHI) and the Lady Health Workers (LHW) program in two rural districts Thatta and Sujawal of the Sindh province, Pakistan (Fig. 1). The population of Thatta and Sujawal districts was 469 504 and 375 193, respectively^13^ and a crude birth rate of 31/1000 population.^14^

The health system in Pakistan is composed of three levels: primary, secondary and tertiary care facilities.^15^^,^^16^ PHC is provided by a Basic Health Unit (BHU) located within a Union Council (UC), which is the smallest administrative unit. This study was implemented in 10 out of 40 BHUs (Fig. 1) with an estimated catchment population of ~ 350 000. Easy access, adequate infrastructure, staffing, availability of the LHW programme and PPHI support were the key factors in the selection of the BHUs.

To form a link between the formal health system and the community, a community-based LHW programme was established by the Government of Pakistan in 1994 to provide health care services at the community level.^17^^,^^18^ Each LHW receives a monthly stipend, serves around 200 households, performs preventive and promotive work related to maternal newborn and child health (MNCH) and family planning and is affiliated with a BHU.

### Implementation of PSBI intervention when a referral to a hospital is not feasible

**Fig. 2:**
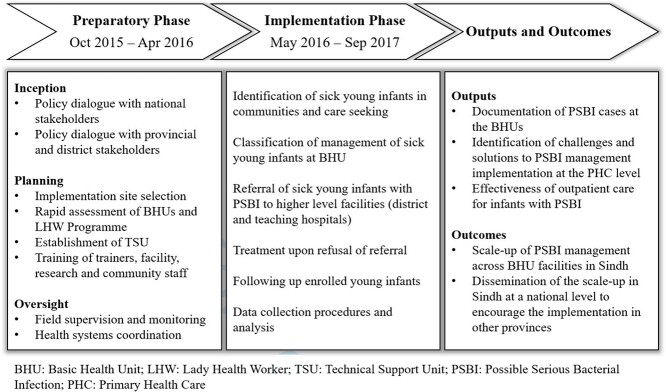
Conceptual framework for implementation research.

Implementation research was carried out between October 2015 and September 2017 (Fig. 2).

### Preparatory phase

#### Policy dialogue

The WHO with the support of the Ministry of Health (MoH) and AKU organized a national level stakeholder meeting for the orientation of PSBI management guidelines and policy dialogue on the potential implementation of the guideline in the country. Stakeholders included policymakers from the MoH, provincial health departments, programme managers, technical experts, members of the paediatrics academia and non-governmental organizations. A consensus was achieved on the management of sick young infants with PSBI signs whose families refused to accept a referral to a hospital with the WHO-recommended treatment on an outpatient basis at selected BHUs in Sindh province (Panel 1).

Panel 1.
PSBI sub-classification and management (8).

**(1) Critical Illness (CI):** Having at least one of the following signs: convulsions, unable to feed at all, no movement at all on stimulation, unable to cry at all, cyanosis, bulging fontanelle. *Management: Give the first dose of pre-referral treatment (Inj ampicillin IM (50 mg/kg per dose) and gentamicin (5–7.5 mg/kg per dose) intramuscularly. If referral is still refused continue twice-daily intramuscular ampicillin and once-daily intramuscular gentamicin for 7 days or until a referral is possible.*

**(2) Clinical Severe Infection (CSI):** Having at least one sign of clinical severe infection (i.e. movement only when stimulated, not feeding well on observation, temperature ≥38°C or ≤35.5°C or severe chest in-drawing).

*Management: Referral for hospitalization after pre-referral treatment.*

*If referral advice is refused, give intramuscular gentamicin 5–7.5 mg/kg once daily for 2 days and twice-daily oral amoxicillin, 50 mg/kg per dose for 7 days on an outpatient basis*

**(3) Severe Pneumonia (SP):** 0–6 days old infants with a respiratory rate of ≥60 breaths per minute.

*Management: Referral for hospitalization after pre-referral treatment. If referral advice is refused, then give twice-daily oral amoxicillin, 50 mg/kg per dose for 7 days on an outpatient basis*

**(4) Pneumonia:** 7–59 days old infant with a respiratory rate of ≥60 breaths per minute.

*Management: Oral amoxicillin, 50 mg/kg per dose twice daily for 7 days on an outpatient basis without referral to a hospital.*

#### Memorandum of understanding between collaborators

Consultative meetings between senior officials of Sindh health department, Sindh LHW Programme and MNCH Programme, PPHI and AKU research team were held to delineate roles of each partner in the implementation (Panel 2), and a memorandum of understanding (MoU) was signed.

Panel 2.
Roles and responsibilities of partners.

**People’s Primary Health Care Initiative (PPHI)**

PPHI is a non-profit organization that works in a public–private partnership with the Government of Sindh and is responsible for managing and delivering services at BHUs in Sindh province. It agreed to lead the implementation of the PSBI guideline in 10 selected BHUs in Thatta and Sujawal districts. A typical BHU deploys a male and a female physician, nurse, dispenser and health technician (paramedic staff). Twenty physicians and 34 paramedical staff served at the 10 selected BHUs. The supply chain was managed and sustained by PPHI. The physicians and nurses were trained in PSBI management guideline and were responsible for identifying infants with PSBI, providing treatment and counselling for follow-up visits and adherence to the guideline.

**Lady Health Worker (LHW) Programme**

LHW programme was responsible to extend support in implementation at the community level, and 179 LHWs and 10 Lady Health Supervisors (LHSs) were linked with

the 10 selected BHUs. During their routine scheduled household visits, the LHWs identified sick young infants with signs/symptoms of PSBI and advised referral to the nearest BHU for further management. LHWs were also responsible for pregnancy surveillance in their respective catchment population. LHW programme also committed to including PSBI guideline in the LHW training curriculum.

#### Establishment and role of technical support unit (TSU)

A TSU was established at AKU to provide technical support, training, collaboration with key partners, supervision and clinical monitoring of the facility staff, including physicians, for quality implementation of the guideline and identification of problems and possible solutions. The TSU members included the study investigators, a study manager and two clinical monitors. The study manager was a paediatrician trained in PSBI management and well versed in the care of young infants. He was responsible for ensuring compliance with protocols at the BHUs and coordinated with the investigators. Also, the TSU employed 10 data collection assistants (DCAs), one for each BHU. DCAs were responsible for data collection and documentation required for the implementation study.

#### Situation analysis

A rapid assessment of the selected BHUs included the assessment of existing infrastructure, staffing, dedicated examination place and operational pharmacy which sourced the essential medicines services. Basic equipment required for the implementation, including weighing scales, stethoscopes, syringes and digital thermometers, were available at the BHUs. Injectable gentamicin and ampicillin, and oral amoxicillin were procured by AKU for the initial few months to avoid stockouts. WHO provided the respiratory rate (RR) timers.

### Building the health system capacity

#### Training

The WHO facilitated a 3-day training of master trainers (TOT) on ‘Managing PSBI in sick young infants up to 2 months of age when a referral is not feasible’. The relevant WHO documents about sick young infants with PSBI when a referral is not feasible^19^ were used for training. Most participants were master trainers of the National Integrated Management of Childhood and Neonatal Illness (IMNCI) programme.^20^ These master trainers subsequently trained the BHU staff of 19 physicians, 12 nurses and 9 health technicians and the TSU field staff in two batches. They were also trained on completing the case reporting forms (CRFs). Also, 170 LHWs and LHSs serving in the catchment population of the selected BHUs were trained on pregnancy and birth surveillance tools, the identification of sick young infants for a referral to nearby BHUs and of thermometers and respiratory timers. Video aids and flip charts in the local language were used, along with clinical practice on sick young infants.

#### Supervision and monitoring, and coordination

The TSU clinical coordinators monitored the BHU staff in the identification and management of PSBI in young infants. They validated randomly 20% of case enrolments either at the BHU or at the household level on the same day. The TSU monitors also conducted weekly households visits to validate the danger signs and compliance with the treatment protocol and mentored the BHU staff as needed. The BHU Physicians organized monthly meetings of the facility and community teams (nurses, LHWs, TSU staff) and discussed various aspects of implementation. The LHS accompanied the LHW during household supervisory visits every month in her catchment area for confirmation of pregnant women registered, births reported, young infants identified with danger signs and referred by the LHW.

The study manager provided oversight and monitored the progress of the study through monthly visits to each BHU. He liaised and discussed implementation challenges with PPHI and LHW programme managers and possible solutions to improve cases detection and the quality of service offered.

### Implementation phase

#### Identification of sick young infants at community and care-seeking

LHWs collected pregnancy and births information from their catchment area to achieve high coverage of the intervention. In some LHW programme uncovered areas, community health workers (CHW) were hired and trained to provide the same services. The LHWs/CHWs were in close liaison with families, traditional birth attendants and community midwives to capture information on births within the first 24 h. They carried out regular community awareness sessions with the DCAs to educate families on danger signs and early care-seeking at BHUs. The sick young infants were identified by the LHW/CHW during routine home visits and referred to the nearest BHU or brought by families to either the LHW/CHW or the BHU (self-referral).

#### Management of sick young infants with PSBI signs at BHU

All sick infants presenting at the BHU were assessed for PSBI signs by the physician. A 7–59 days old infant with only fast breathing (≥60 breaths per minute) was managed without referral to a hospital. Infants with other PSBI signs were referred to the district hospital after administering a pre-referral antibiotic dose (Panel 1). If the family refused referral, PSBI was further classified and managed according to the WHO PSBI guideline (Panel 1).

The BHU provided ambulance service at minimal charges. A referral card detailing the condition (vitals, weight and presence of danger signs) with documentation of the pre-referral antibiotic dose (injectable gentamicin) was given to the families. For families who refused referral despite extensive counselling, simplified antibiotic treatment was initiated on an outpatient basis after written consent was obtained (Panel 1). The nurse administered the first dose of intramuscular gentamicin and oral amoxicillin at the BHU. The second dose of oral amoxicillin was given at home by the mother. The family was asked to return for a follow-up the next day at the BHU to receive the second dose of injectable gentamicin, whereas the remaining dosages of oral amoxicillin were administered at home to complete 7 days treatment course.

#### Follow-up of sick young infants

Enrolled infants were followed at the BHU on Days 4 and 8 after the initiation of treatment to monitor the clinical response, treatment adherence and adverse effects. If the infant’s condition worsened, he/she was categorized as treatment failure and referred to the district hospital. If the families did not return for the second dose of intramuscular injection, the study DCA would coordinate with the LHW/CHW to visit their homes and encourage them to visit BHU for treatment. A PSBI card with patient information, classification, treatment, and referral was developed to strengthen the follow-up.

#### Data collection and analysis

All clinical and follow-up data were collected on paper-based instruments. The TSU employed 10 DCAs, one for each BHU for collecting data on the infant seeking care at the BHU facility. The DCAs were qualified lady health visitors/midwives who were trained in WHO PSBI guidelines. These data collectors collated information on the general condition including weight, breastfeeding assessment, presence of jaundice, cord care and presence of danger signs for PSBI. The outcome of the BHU visit including classification of PSBI, acceptance or refusal to referral and ambulatory treatment regimens were documented. CRFs were developed for LHWs post-natal home visits to evaluate and recognize sick newborns (danger signs) and refer them to the BHU facility for case management. In addition, newborn mortality data were also collected. Follow-up information on treatment, adherence, compliance and outcome were recorded by study staff. A follow-up form on Day 14 captured information on all infants that accepted referral or were hospitalized. Information on vital status was finally collected at Day 59 for all participating infants. Qualitative data were collected to identify challenges and enablers encountered during the implementation. We conducted in-depth interviews with the physicians and administrative staff of BHU and focus group discussions with the LHWs and the community members. In addition, we took notes of observations during the scheduled monitoring visits. Before data entry, all forms were checked for completeness and consistency and were coded. The data were entered twice using Microsoft FoxPro and analysed using STATA v.15. Descriptive analysis was performed using frequencies and the proportion of sick young infants identified with PSBI subcategories referred to a district hospital, refused referral and were treated at BHU, followed up, adhered and completed treatment, failed treatment, died and developed adverse outcomes and those cured.

### Ethical consideration

This study was approved by the AKU Ethical Review Committee (ERC Number: 3936-Ped-ERC-15) and WHO Research Ethics Review Committee. Caregivers of infants provided informed, signed consent before enrolment in the study. Those who were unable to sign provided a thumb impression to express consent.

## Results

During the study period of October 2015 and September 2017, 17 600 live births were recorded in the study area. Study BHUs were visited by 5100 young infants and 1860 (36.5%) were identified by a physician with signs of PSBI (Table 1). Assuming a 10% incidence of PSBI^8^^,^^10^^,^^12^ among all live births, we expected 1760 young infants with PSBI signs within the first 2 months of life. We estimated that 10% of young infants came from outside the study catchment area. Thus, the coverage of the treatment was 95%. Of the 1860 PSBI cases, 5.3% (98) were classified as having a critical illness (CI), 26.6% (495) as clinical severe infection (CSI), 18% (344) young infants 0–6 days had severe pneumonia and 49.6% (923) young infants 7–59 days of age had pneumonia (Table 1). Sixty per cent (1113/1860) of young infants with a sign of PSBI were brought to BHUs by families themselves (Table 2).

**Table 1:** Characteristics of young infants 0–59 days who visited the study BHUs

**Characteristics**	**n/N (%)**
Age in days	
0–6 days	1289/5100 (25.3)
7–28 days	2260/5100 (44.3)
29–59 days	1551/5100 (30.4)
Sex	
Male	2647/5100 (51.9)
Female	2453/5100 (48.1)
Classification of illness	
Possible Serious Bacterial Infection	1860/5100 (36.5)
*Critical Illness*	*98/1860 (5.3)*
*Clinical Severe Infection*	*495/1860 (26.6)*
*Severe Pneumonia (fast breathing in 0–6 days of age)*	*344/1860 (18.5)*
*Pneumonia Fast Breathing in 7–59 days of age*	*923/1860 (49.6)*
Local Infection	673/5100 (13.2)
Infection unlikely	2567/5100(50.3)

**Table 2:** Referral acceptance and treatment adherence of infants 0–59 days of age with signs of PSBI (*n* = 1860)

	**PSBI classification**			
**Parameters**	**Fast breathing 7–59 days n (%)**	**Fast breathing 0–6 days n (%)**	**Clinical severe infection n (%)**	**Critical illness n (%)** ^§^
Number of young infants identified with PSBI at BHUs	923	344	495	98
Number brought by families to the BHU (self-referral)	567 (61.4)	200 (58.1)	302 (61.0)	44 (44.9)
The number identified by the LHW in the community and referred to the BHU	356 (38.6)	144 (41.9)	193 (39.0)	54 (55.1)
Number of infants accepted referral to a hospital	0 (0.0)	9 (2.6)	55 (11.1)	92 (93.9)
Number of infants treated at a BHU with a simplified antibiotic regimen on an out-patient basis	923 (100.0)	335 (97.4)	440 (88.9)	6 (6.1)
**Adherence to treatment in young infants treated with a simplified antibiotic regime at BHU**				
Fully adherent^*^	708/923 (76.7)	238/335 (71.0)	305/440 (69.3)	1/6(16.7)
Partially adherent^†^	71/923 (7.7)	28/335 (8.4)	63/440 (14.3)	1/6(16.7)
Non adherent^‡^	144/923 (15.6)	69/335 (20.6)	72/440 (16.4)	4/6 (66.7)
**Follow-up of infants who accepted treatment at BHU**				
Number completed all follow-up visits^**§**^	596/923 (64.6)	206/335 (61.5)	170/440 (38.6)	1/6(16.7)
Number successfully followed-up on day 4	758/923 (82.1)	272/335(81.2)	349/440 (79.3)	5/6 (83.3)

^*^Fully adherent: for critical illness seven gentamicin+14 ampicillin injections, for clinical severe infection two injections of gentamicin +14 oral amoxicillin doses, for fast breathing 14 oral amoxicillin doses

^†^Partially adherent: critical illness five gentamicin+10 ampicillin, for clinical severe infection one injections of gentamicin +10–13 oral amoxicillin doses, for fast breathing 10–13 oral amoxicillin doses

^‡^Non-adherent: not fulfilling the definition of full or partial adherence.

^§^For critical illness the complete follow-up is on days1–8 and 14, for the clinical severe infection the complete follow-up is on days 2, 4, 8 and 14 and for fast breathing, the complete follow-up is on day 4, 8 and 14.

Infants 7–59 days of age with fast breathing pneumonia were prescribed oral amoxicillin for 7 days. The first dose was administered at a BHU. They were followed up at home on Days 4, 8 and 14 by the study LHW/CHW. Complete adherence to the treatment was 76.7% (708/923) and 93.8% (866/923) of infants recovered on oral amoxicillin (Tables 2 and 3). Young infants with CI, CSI and severe pneumonia (fast breathing in infant 0–6 days of age) who required referral to a hospital were identified in 50.3% (937/1860) of all PSBI cases (Table 2). Families of 83.3% (781/937) sick young infants refused referral advice and hence, were treated on an outpatient basis with a simplified antibiotic regimen (Panel 2).

**Table 3:** Treatment outcomes of treated patients by the place of treatment

**PSBI classification**	**Hospital treatment (Accepted referral)**		**Outpatient treatment (Refused referral)**		
	**Number treated (*n* = 156) n (%)**	**Died by day 14 (*n* = 12) n (%)**	**Number treated (*n* = 1704) n (%)**	**Clinical treatment failure (*n* = 118) n (%)**	**Died by day 14 (*n* = 6) n (%)**
Fast breathing 7–59 days	0 (0·0)	0 (0·0)	923 (100·0)	57 (6·2)	0 (0·0)
Severe pneumonia (0–6 days)	9 (2·6)	1 (11·1)	335 (97·4)	24 (7·2)	1 (0·3)
Clinical severe infection	55 (11·1)	5 (9·1)	440 (88·9)	34 (7·7)	4 (0·9)
Critical illness	92 (93·9)	6 (6·5)	6 (6·1)	3 (50·0)	1 (16·7)

^*^The six deaths are not included in the treatment failure

Of the 98 young infants identified with signs of CI, 93.8% (92/98) families accepted referral to higher health facilities, six families refused referral despite repeated counselling and were treated with injectable ceftriaxone once a day daily for 7 days at the BHU. All were followed up till Day 14. Most (80.6%, 79/98) recovered, 12% (12/98) were still sick and seven died with a case fatality rate (CFR) of 7.1% (7/98) (Table 3). CSI was identified in 52% (495/937) of sick young infants and only 11.1% (55/495) families accepted the referral. The remaining were treated at a BHU with the simplified antibiotic regime on an outpatient basis (Panel 1). The treatment success was 85.5% (376/440). Four deaths were reported at Day 14 follow-up (Table 3). In the 0–6 days old young infants with severe pneumonia, 97.4% (335/344) refused referral to a hospital and were treated at a BHU on an outpatient basis (Panel 1) Only 7.2% (24/335) failed treatment and one death was reported on Day 14 (Table 3).

Forty-five per cent (70/156) of families who accepted a hospital referral went to a public hospital (Table 4). Twelve infants died, while 12 were still found to be ill at Day 14 follow-up (Table 3).

**Table 4:** Place and type of treatment in those who accepted referrals from BHU (*n* = 156)

	**Fast breathing 0–6 days (*n* = 9) n (%)**	**Clinical severe infection (*n* = 55) n (%)**	**Critical illness (*n* = 92) n (%)**
Public hospital	6 (66·7)	25 (45·4)	39 (42·4)
Private hospital/clinics	3 (33·3)	30 (54·6)	53 (57·6)
Treatment received			
Injectable including antibiotics	9 (100·0)	55 (100·0)	92 (100·0)
Other oral medications	3 (33·3)	36 (65·5)	66 (71·7)
Oxygen	1 (11·1)	9 (16·4)	20 (21·7)

We identified several challenges that were encountered during the implementation research within our study setting, ranging from compliance to guidelines by health care providers, documentation of cases, motivation of CHW and lack of compliance by family members, to which we tried to identify solutions to rectify these challenges (Table 5).

**Table 5:** Challenges and solutions encountered during the study implementation period

Underlying factors for non-compliance and poor performance	Proposed Actions and Solutions	Outcomes Achieved
**Challenge: Health Care Providers performance and non-compliance to PSBI to guidelines.**		
Lack of knowledge and inexperience in executing the IMNCI/ PSBI guidelines	At the initial phase, TSU staff (LHVs) who were well versed with the case management of sick young infants supported the physicians in the assessment and classification of PSBI.	Enhanced the competency of the health care providers and consequently led to adequate compliance to PSBI guidelines.
	Training of PPHI Physicians and Nurses in IMNCI guidelines	Improved confidence of physicians in recognition and management of sick younginfant.
	Refresher training and on-site supervision by TSU clinical coordinators	
Low performance due to high volume outpatient clinics and regular engagement in supplementary polio immunization activities (SIAs)	Motivation and mentoring visits by PPHI Managers.	Improved performance and compliance of the BHU Staff.
	Initiation of performance-based incentives by PPHI to inculcate a healthy competitive environment	
**Challenge: Physicians reluctance to administer I/M intramuscular injections to febrile infants.**		
This clinical practice adapted from peers physicians during clerkship following graduation from medical school.	A one-day technical seminar organized for BHUs physicians. AKU senior faculty members address concerns of physicians and share the standard clinical practice guidelines of antibiotic administration.	Change in practices of physicians observed.
	Regular reinforcement and counselling by the TSU and PPHI clinical coordinators and monitors.	Improved adherence to the treatment guidelines.
**Challenge: Poor documentation of case summary of young sick infants with PSBI**		
Unaccustomed to record- keeping	The TSU staff carried out the documentation in the initial period of the implementation. Later the physicians took up the responsibility.	Documentation improved by facility staff
**Challenge: Poor LHWs performance, motivation**		
Demotivation and poor performance of the LHW due to; Poor health system poor governance.	TSU carried out dialogues with the LHW program and feedback provided to the LHW program coordinator in the monthly district health management meetings.	Improvement in the LHW performance reflected by the increased referrals of PSBI cases to BHUs.
Delay in monthly salaries and replenishment of essential LHW delivery commodities (ORS, zinc and oral amoxicillin).	Joint field monitoring visits by the Lady Health Supervisors and TSU clinical coordinators to validate the data on pregnancy surveillance, PSBI cases and referrals.	
The frequent engagement of LHWs in Polio immunization campaigns.	Feedback of monitoring visits provided to LHWs and counselling to improve performance.	
**Challenge: Family noncompliance to injectable and oral antibiotics treatment**		
Families declined injectable antibiotics treatment as they were afraid of injections and sharp needles.	LHWs visited households to reinforce completion of treatment. They counselled families on the importance of the prescribed duration of treatment and ensured them of the safety of injections.	These actions mitigated the issue of non-adherence to injectable/oral antibiotics treatment and follow-up compliance.
Parents would stop oral antibiotics if the child’s symptoms improved earlier than 7 days.	One of the reasons identified was the volume of oral syrup amoxicillin 125mg/5ml. The syrup volume was large and inconvenient to administer to small infants by parents. Syrup was replaced with oral amoxicillin drops (125 mg/0.8 ml) to reduce the liquid volume of the amoxicilin.	
Day 2 follow up compliance at BHU was poor.	The importance of follow-up was reiterated in the community awareness sessions conducted by LHWs	
**Challenge: Documentation of PSBI cases**		
The data entry software system (Dashboard) established by PPHI did not capture PSBI cases	The TSU assisted PPHI in designing of PSBI indicators documentation in Dashboard.	PSBI cases captured in the dashboard used to monitor the monthly performance of BHUs for PSBI coverage.

LHV: Lady Health Visitor; ORS: Oral Rehydration Solution.

## Discussion

### The main findings of this study

It is feasible to implement WHO PSBI management guidelines in the existing health system settings when a referral is not feasible. We achieved high coverage of 95%, low treatment failure (118/1704; 6.92%) and low CFRs (0.35%) in the existing public health system. An overwhelming majority in our study who needed a referral to a hospital refused and accepted a simplified outpatient antibiotic regimen near their homes, despite the severity of the disease.

### What is already known on this topic

Our treatment coverage rate was higher than those reported in Malawi (63.8%),^21^ Lucknow in India (53%)^22^ and Kushtia in Bangladesh (31%),^23^ but was comparable to a study in Mekelle, Ethiopia (87%).^24^ Majority of families sought care themselves, which underscores the importance of effective community mobilization by CHWs/LHWs in care-seeking behaviours and their role in the health systems in low-middle income countries (LMICs).

The high refusal rates in our study settings are similar to those reported at other implementation sites, such as 97% in Nigeria,^25^ 83.3% in Bangladesh^26^ and >90% in Malawi.^21^ However, we observed a much higher hospital referral acceptance among families whose young infants had a critical illness. The reasons cited for refusal to accept referral advice across the sites were similar to ours, which included lack of access to care, distance to the facility, cost of treatment and poor quality of the referral facility care.^23^^,^^26^^,^^27^ A 10th of families in our study of young infants with CSI signs accepted referral to a hospital, while majority were treated at the BHU with high treatment success (90%) and low CFR (0.9%). Effectiveness of the simplified antibiotic regimens used is well documented in large trials in several countries and recommended by WHO.^8^ PSBI implementation research in other LMIC countries also showed a high refusal to referral, increased compliance to simplified antibiotic guidelines and low fatality rates. In Bangladesh, among those who accepted outpatient treatment, 80% returned for a second dose of gentamicin.^26^ Similarly, in Nigeria, nearly all (96%) treated at PHC centres received the correct dosage of gentamicin and amoxicillin with a low clinical treatment failure of 1.3%.^25^

Twelve deaths occurred among all PSBI cases that were treated at a hospital (7.7%), compared with only six (0.4%) those treated on an outpatient basis. The higher CFR in hospitalized PSBI cases could be due to the severe illness, delay in care-seeking or sub-optimal quality of care received at the hospital. This CFR was similar to that reported in a systematic review from low- and middle-income countries; 9.8% from both prospective and retrospective studies and 7% in only prospective studies.^3^ However, the low CFR of young infants with PSBI treated on an outpatient basis with simplified antibiotic regimens in our study is noteworthy and supports an effective intervention implemented with high coverage in a programme setting.

### What this study adds

The young infants 7–59 days of age with fast breathing only were treated safely with oral amoxicillin in outpatient settings without a referral, which was not the usual practice before the implementation of the PSBI guideline. High treatment adherence to oral amoxicillin and a high recovery rate was documented. However, on several occasions, families stopped the antibiotics and did not complete the entire course if the symptoms improved before the completion of 7 days of treatment. In several cases, administration of a large volume of amoxicillin suspension was not accepted by young infants, which partly contributed to nonadherence. With the procurement and introduction of oral amoxicillin drops with reduced volume dose, adherence improved. However, our adherence of 77% was <98% reported from Ethiopia, and their clinical treatment success of 99%^24^ was also higher than our 94%.

We found that simple outpatient antibiotic regimens were effective in programmatic settings with high treatment success rates for fast breathing and CSI despite concerns about substantial antimicrobial resistance to commonly used antibiotics.^28–30^ We believe increasing the coverage of PSBI management guidelines across the country can have a manifold impact on newborn health outcomes, including improving case management and clinical practice, rational use of antibiotics, reduction in antimicrobial resistance due to the injudicious use of broad-spectrum antibiotics by registered and unregistered health care providers in the community.^31–33^

### Strengths and limitations of this study

Our research had several strengths. The study was implemented within a reasonably well-equipped and functional existing health system. The technical support by TSU ensured adequate competency in case management, supervision and monitoring to improve the performance of health care providers. PPHI, our implementing partner, and the TSU’s excellent engagement and effective strategies were instrumental in overcoming the challenges encountered. However, we also faced several operational hurdles including the underutilization of PHC by the community due to unsatisfactory services provided by the PHC staff. The PHC staff were inexperienced in case management and initially refused to administer intramuscular injections to febrile infants. These operational challenges were overcome with training, supervision, mentoring of the health care providers, community mobilization and engagement with the LHW program. The supply chain sustainability was another obstacle with frequent stock out of essential commodities, which was mitigated by procurement through project funds. The LHW coverage was inconsistent and frequent incentivized assignments for polio campaigns led to absenteeism that slowed the implementation process at the community level. The TSU staff, DCAs supported the LHWs/CHWs and carried out pregnancy surveillance, household visits and community sessions in their absence. Finally, we have used some assumptions for the incidence of PSBI from other published data and the proportion of patients coming from outside the catchment area, which may not be accurate. A higher incidence assumption would result in lower treatment coverage, whereas a lower assumption for those coming from outside the catchment area would result in higher treatment coverage.

We learned a few lessons that are useful for future scale-up. A strong linkage and engagement between the community, the LHW programme and implementers will be crucial to improve prompt care-seeking and ensure good coverage of treatment with low fatality. Moreover, adherence to the management guideline by PHC staff and high LHW coverage in the catchment population is essential for a successful scale-up.^27^ This provides an opportunity to improve the quality of care for maternal and newborn care. For effective implementation and sustainability, the government and implementing partners need to pledge their continuous support for capacity building, retention of health worker skills for quality of care at BHUs and sustained supply of necessary commodities. The health management information systems do not include PSBI case management and treatment outcomes, so its integration into routine measurement is essential for successful implementation. The TSU active participation was critical in ensuring capacity building, monitoring, strengthening linkages among all partners and quality assurance. Some kind of technical support will be needed for the PHC staff during scale-up to assist them. Adherence to follow-up was high due to regular telephonic contacts with the family and household visits made by the study staff. Hence a strong network of LHWs/CHWs, effective community mobilization and a strong mechanism of follow-up would be required for high coverage and compliance to treatment.

## Conclusions

To conclude, this implementation research showed that it is feasible to implement PSBI management guideline at the PHC level where referral is not feasible or not possible. The implementation research highlighted several challenges that were overcome by collaborative efforts of implementing partners and technical support of TSU. This is a complex intervention, and the scale-up of the implementation would require a functional health facility, strong commitment, sustained support and effort from the government with continued technical assistance and oversight across PHC centres.

## Data Availability

The data of the study are available upon request keeping in view institutional ethical polices by emailing to corresponding author.

## Notes

**Registration:** The study is registered on the Australian New Zealand Clinical Trials Registry as ACTRN12617001373369. **Shabina Ariff**, Associate Professor **Sajid Bashir Soofi**, Professor **Zamir Suhag**, Director Research **Suhail Chanar**, Manager Research **Maria Bhura**, Research Coordinator **Zaib Dahar**, Director Child Health **Imran Ahmed**, Senior Manager, DMU

**Ali Turab**, Senior Instructor Research **Atif Habib**, Assistant professor Research **Yasir Bin Nisar**, Medical Officer **Samira Aboubaker**, Ex Medical officer **Steve Wall**, Technical Advisor **Abdul Wahab Soomro**, CEO **Shamim Ahmad Qazi**, Ex Medical Officer **Rajiv Bahl**, Coordinator Newborn Health & MCA **Zulfiqar A Bhutta**, Professor & Founding Director
